# Molecular dynamics simulation of dopamine and ascorbic acid amid tetrafluoroborate 1-butyl-3-methylimidazolium compared to conventional solvents

**DOI:** 10.1186/1753-6561-8-S4-P90

**Published:** 2014-10-01

**Authors:** Lucas Santos, Moacyr Comar

**Affiliations:** 1 Universidade Federal de São João Del-Rei, Divinópolis, Brazil

## 

Dopamine (DA) is an important neurotransmitter in the Central Nervous System (CNS) of mammals being found in significant quantities in the brain. In a low concentration is associated with Parkinson's disease [1], which makes it important detection. However, a major problem in determining DA is its joint resolution with coexisting species such as ascorbic acid (AA).

In traditional solid electrodes, AA is oxidized at a potential close to that of DA, resulting in an overlapping voltammetric response [2]. Therefore, improving the selectivity of the monitoring techniques of AD has been the focus of much research. [3] Thus, ionic liquids at ambient temperatures (RTILs) tetrafluoroborate and 1-butyl-3-methylimidazolium (BMI.BF4) can be used as electrolytes for different electrochemical reactions, which have important properties, the selectivity of them, making the technique voltammetric more efficient.

Our focus as this work is to analyze the behavior of these molecules (DA and AA) amid BMI.BF4 compared to conventional solvents, using a method known as Molecular Dynamics (MD), being a powerful tool for obtaining properties structural and thermodynamic properties of these substances.

MD simulations were carried out for systems in DA [BMI.BF4] and AA [BMI.BF4]. For comparison, simulations were also carried out in water. All simulations were performed using the AMBER 9 package. We also used PTRAJ to perform analyses on the data we collected, such as calculating density, total energy, RDFs and self-diffusion coefficients, and we used xmgrace and gnuplot to aid us in visualizing the data.

As part of evaluating the interference between molecules (AA and DA), we show the results of analyzes of AA. In this case the average density is 1.0768 cc / mol, while the water density is 1.027 cc / mole. As the total energy of the two systems have different values and constants (-19.5 kcal / mol in the midst of water and -12.5 kcal / mol among LI) along the dynamics. This difference in energy is given by unfavorable interactions that AA has with the molecules of the LI. For a better understanding of the systems were calculated the radial distribution functions for each of statepoints. The results presented in both solvent were similar, demonstrating that the AA is added in both LI well as for water, ie they have good solvation.

Regarding the results of diffusivity, the 298K and 1 atm the coefficient of self-diffusion (MSD) showed lower values compared to the water in the same conditions. This result reveals the MSD in a higher viscosity of the LI and so may be an important property for separation of molecules (AA and DA) and therefore important in electrochemical process.
